# Evaluation of acute, 28-day, 13-week repeated dose oral toxicity and genotoxicity of a herbal extract (HemoHIM G) from *Angelica sinensis, Ligusticum chuanxiong,* and* Peaonia lactiflora*

**DOI:** 10.1007/s43188-024-00227-2

**Published:** 2024-03-16

**Authors:** Su-Bin Bak, Hansol Choi, Gyoung-Deuck Kim, Ju Gyeong Kim, Da-Ae Kwon, Ha-Young Kim, Dong-Won Son, Jang-Hun Jeong, Byung-Woo Lee, Hyo-Jin An, Hak Sung Lee

**Affiliations:** 1 Food Science R&D Center, Kolmar BNH Co., Ltd., 61, Heolleung-ro 8-gil, Seocho-gu, Seoul Republic of Korea; 2 Biotoxtech Co., 53, Yeongudanji-ro, Ochang-eup, Cheongwon-gu, Cheongju-si, Chungcheongbuk-do 28115 Republic of Korea

**Keywords:** HemoHIM G, Functional food, Acute toxicity, Repeat dose toxicity, Genotoxicity

## Abstract

HemoHIM G is a functional food ingredient composed of a triple herbal combination of *Angelica sinensis*, *Ligusticum chuanxiong*, and *Paeonia lactiflora*, to improve impaired immune function. Considering the pharmacological benefits of its constituent herbal components, HemoHIM G is anticipated to have various health benefits; however, its toxicity has not been thoroughly evaluated. Here, we conducted a comprehensive study to assess the safety of HemoHIM G in terms of acute oral toxicity, 13-week repeat-dose toxicity, and genotoxicity. In the oral acute toxicity study, Sprague–Dawley rats were orally administered a single dose of HemoHIM G at 5000 mg/kg/day, the limit dose for the acute study. No abnormal findings or adverse effects were observed in this study, as confirmed by gross pathology. A 13-week repeated-dose toxicity study was conducted with HemoHIM G at doses of 1250, 2500, and 5000 mg/kg/day to examine the subchronic toxicity in both male and female rats after 28 days of dose-range finding study. No test substance-related clinical signs or mortality was observed at any of the tested doses. Gross pathology, hematology, blood chemistry, and histopathology were within normal ranges, further supporting the safety of HemoHIM G. Therefore, the NOAEL of HemoHIM G was considered to be at 5000 mg/kg/day for both sexes of rats. Bacterial reverse mutation tests, a chromosome aberration test in human peripheral blood lymphocytes, and a mouse micronuclei test were conducted to identify the potential genotoxicity of HemoHIM G. HemoHIM G is non-mutagenic and non-clastogenic. Collectively, these findings provide valuable evidence for the safe use of HemoHIM G as a functional food ingredient.

## Introduction

In traditional Oriental medicine, many herbs and herbal formulations have gained recognition for their remarkable ability to enhance well-being, bolster the body’s natural defenses, and foster longevity [1] Combining multiple medicinal herbal extracts represents a novel strategy to overcome the drawbacks of single herbal extracts by improving their efficacy and safety [2, 3]. HemoHIM G is a functional food ingredient composed of a triple herbal combination of *Angelica sinensis*, *Ligusticum chuanxiong*, and *Paeonia lactiflora*, to improve impaired immune function.

Herbal extracts that constitute HemoHIM G are believed to exert beneficial effects when used alone or in combination. *Angelica sinensis* is a medicinal plant with a rich history of use in traditional Chinese medicine. It is commonly used in herbal remedies to treat female ailments, menstrual irregularities, and blood replenishment [4]. Detailed chemical analysis revealed its diverse composition, including phthalides, organic acids, polysaccharides, flavones, coumarins, and inorganic elements [5]. Phthalides, organic acids, and polysaccharides have received increasing attention because of their extensive investigation and demonstrated potent pharmacological effects.

*Ligusticum chuanxiong* is highly regarded in China because of its numerous health benefits. It is traditionally believed that the regular consumption of *L. chuanxiong* promotes overall health and effectively prevents cerebrovascular diseases [6–9]. Extensive phytochemical investigations have revealed the presence of volatile oils, phenols, alkaloids, and polysaccharides in *L. chuanxiong* [10, 11].

*Paeonia lactiflora* is a well-known medicinal plant recognized for its anti-inflammatory effects and the ability to modulate immune cells and autoimmune diseases [12]. The roots of *P. lactiflora,* extensively used in traditional medicine across Korea, China, and other South Asian countries, contain various bioactive components, such as albiflorin, paeoniflorin, paeonol, and phenolic compounds [13–15].

Given the remarkable therapeutic properties of these herbal constituents, HemoHIM G shows great promise as a prospective therapeutic intervention and preventive approach for many diseases. While these individual plants have been extensively studied in traditional oriental medicine, limited research has been conducted on their combined formulation, HemoHIM G. Therefore, a thorough investigation is warranted to explore the potential effects of HemoHIM G on human health and assess any associated risks. Thus, this study aimed to assess the acute and subacute toxicity of orally administered HemoHIM G in rats, and its in vitro and in vivo genotoxicity. This study provides valuable insights into the safety of HemoHIM G as a potential therapeutic agent.

## Materials and methods

### Test substances

HemoHIM G (Lot No. 2100001) was prepared by concentrating extract under reduced pressure to achieve an optimal concentration. No additional additives were introduced during this process. The concentrated extract was then transformed into a powdered form through lyophilization. HemoHIM G extract is checked and standardized for its consistency with chlorogenic acid (more than 80% of 0.216 mg/g) derived from *Angelica sinensis* and *Ligusticum chuanxiong*, and paeoniflorin (more than 80% of 2.869 mg/g) derived from *Paeonia lactiflora*, as marker substances, manufactured by Kolmar BNH Co. Ltd. (Sejong, Korea). The sample for safety test contains chlorogenic acid 0.216 mg/g, and paeoniflorin 2.869 mg/g. In addition to the marker substances, ferulic acid and (*Z*)-ligustilide from *Angelica sinensis* and *Ligusticum chuanxiong*, senkyunolide derivatives such as senkyunolide A and H from *Ligusticum chuanxiong*, and paeoniflorin, albiflorin, and gallic acid from *Paeonia lactiflora* were confirmed to be contained in HemoHIM G. The formulation was prepared immediately before administration, on the same day it was intended to be administered.

### Animals and husbandry

All rats (Crl:CD(SD), 6 weeks old) for this studies were obtained from Orientbio Inc. (Seongnam, Korea). Environmental conditions in the animal room were maintained as follows: temperature = 19–25 °C, relative humidity = 30–70%, air exchange rate 10–15 changes/h, and light/dark cycle = 12 h/12 h. Variations in these conditions had no effect on the study outcomes.

This study was conducted in accordance with the following Good Laboratory Practice Regulations: “Good Laboratory Practice Regulation for Nonclinical Laboratory Studies”, Notification No. 2018–93, Ministry of Food and Drug Safety, Republic of Korea (Nov. 21, 2018); “OECD Principles of Good Laboratory Practice”, Organization for Economic Co-operation and Development (OECD), ENV/MC/CHEM(98)17 (as revised in 1997).

This study was conducted at Biotoxtech Co., Ltd. (Cheongju, Korea), which received full accreditation from the Association for Assessment and Accreditation of Laboratory Animal Care International (AAALAC International) in 2010. This study was reviewed and approved by the Institutional Animal Care and Use Committee (IACUC) of Biotoxtech Co., Ltd. based on Animal Protection Act of Republic of Korea (Enactment May 31, 1991, No. 4379, Revision Feb. 11, 2020, No.16977) (Approval No.: 220298).

### Acute oral toxicity

An acute oral toxicity study was conducted in accordance with Organization for Economic Cooperation and Development (OECD) Guideline 423 following the application of good laboratory practice (GLP) [16]. The acute toxicity of HemoHIM G was assessed in male and female SD rats via oral gavage, with the test substance dissolved in water for injection and administered at the dose limit of the preliminary study (5000 mg/10 mL/kg of body weight). All animals were fasted overnight (16 h) (water *adlibitum* with no feed) before administrating the test substance. All animals were observed for mortality, morbidity, and signs of toxicity (clinical signs) at 30 min, 1, 2, 4, and 6 h after dosing on day 0 and once daily thereafter for 14 days. Body weights were recorded prior to dosing on days 0, 2, 4, 8, and 15. At the end of the 14-day observation period, necropsy and gross pathological examinations were performed.

### 28-Day repeated dose oral toxicity

A repeated-dose oral toxicity study was conducted in accordance with the OECD Guideline 407 following the application of GLP [17]. The doses were administered orally to SD rats for 28 consecutive days to assess of any toxic effects. The test substances were weighed, suspended in water, and administered to rats through the oral (gavage) route using a disposable syringe with a rat intubation cannula at graduated dose levels of 1250 mg/kg/day for low dose (G2), 2500 mg/kg/day for mid-dose (G3), and 5000 mg/kg/day for high dose (G4). The rats in the control group (G1) received water alone. The administered dose volume was 10 mL/kg/day. Each group consisted of five rats of each sex. Vehicle or test formulations were administered to each rat group once daily for 28 consecutive days. The animals were observed twice daily for mortality/morbidity and once daily for cage-side clinical signs. Detailed clinical examinations were performed once prior to the initiation of treatment and thereafter at weekly intervals and the end of the treatment and recovery periods. The rats were observed once per week for changes in body weight and feed consumption. Hematological and clinical chemistry investigations were performed at the end of the treatment and recovery periods.

### 13-Week repeated dose oral toxicity

A repeated-dose oral toxicity study was conducted in accordance with OECD Guideline 408 following the application of GLP [18]. The doses were administered orally to SD rats for 13 consecutive weeks, followed by a 28-day recovery period to assess the reversibility of any toxic effects. The test substance was weighed, suspended in water, and administered to rats through the oral (gavage) route using a disposable syringe with a rat intubation cannula at graduated dose levels of 1250 mg/kg/day for low dose (G2), 2500 mg/kg/day for mid-dose (G3), 5000 mg/kg/day for high dose (G4), and high dose recovery groups (G4R). The rats in the control group (G1) and control recovery group (G1R) received water alone. The administered dose volume was 10 mL/kg/day. Each group consisted of 10 rats of each sex. Vehicle or test formulations were administered to each rat group once daily for 13 consecutive weeks. The animals were observed twice daily for mortality/morbidity and once daily for cage-side clinical signs. A detailed clinical examination was performed once prior to the initiation of treatment, thereafter at weekly intervals, and the end of the treatment. The rats were observed once per week for changes in body weight and feed consumption. Hematological and clinical chemistry investigations were performed at the end of treatment.

### Bacterial reverse mutation assay

An in vitro bacterial reverse mutation assay was conducted in accordance with OECD Guideline 471 following the application of GLP [19]. In preliminary cytotoxicity assay, TA1537, TA1535, TA100, TA98 of *Salmonella typhimurium* strain were treated with the test substance at the concentrations of 313, 625, 1250, 2500, and 5000 μg/plate both in the presence (S9 mix) and absence of metabolic activation system. Vehicle and positive controls were maintained concurrently with the treatment groups. Based on the results observed in the preliminary cytotoxicity assay, 5000.0 μg/plate was selected as the highest concentration for mutagenicity assay. Mutagenicity assays were performed using the TA1537, TA1535, TA98, and TA100 strains of S. typhimurium and the WP2uvrA strain of E. coli. The bacterial strains were treated with the test substance at 313, 625, 1250, 2500, and 5000 μg/plate in the presence (S9 mix) and absence of a metabolic activation system.

### In vitro mammalian chromosomal aberration assay

An in vitro mammalian chromosomal aberration assay was conducted in accordance with OECD Guideline 473 following the application of GLP [20]. Based on the preliminary cytotoxicity assay results, chromosome aberration assay was conducted using three different concentrations of test substance i.e., 78, 156, 313, and 625 μg/mL in the presence and absence of a metabolic activation system. Benzo[a]pyrene (with the metabolic activation system S9) and mitomycin C (without the metabolic activation system S9) were used as clastogenic positive controls. Chinese hamster lung cell (CHL/IU cell) were cultured using Eagle’s minimum essential medium supplemented with 10% FBS, 1% penicillin–Streptomycin, in CO2 incubator, at 37 ± 1 °C and 5 ± 0.5% CO_2_. These cultures were exposed to different concentrations of test substances for short-term (6 h) and continuous (24 h) exposure. In short-term exposure, after 6 h of treatment, culture media with test substance was replaced with fresh medium and further incubated for 18 h at 37 ± 1 °C and 5 ± 0.5% CO_2_. For continuous exposure, cultured cells were treated with different concentrations of test compounds for 24 h. After 24 h, cultures from the short-term and continuous exposure groups were harvested and processed for slide preparation. The slides were stained with Giemsa stain (3%, v/v). Slides were observed in the order of short-term and continuous treatments. Chromosomal aberrations were classified as structural aberrations, numerical aberrations, etc.

### Mammalian bone marrow erythrocyte micronucleus assay

An in vivo mammalian bone marrow erythrocyte micronucleus assay was conducted in accordance with OECD Guideline 474 following the application of GLP [21]. Micronucleus tests were conducted at doses of 1250, 2500, and 5000 mg/kg. The dose levels for the micronucleus test were selected based on a dose-range finding study. For the micronucleus test, HemoHIM G was orally administered to SD rats at a dose volume of 10 mL/kg for 2 days, with an interval of approximately 24 h. Animals in the positive control group received a single dose of cyclophosphamide orally at 20 mg/kg/day before bone marrow collection. Approximately 24 h after dosing, all animals were euthanized and both femur bones were collected from each animal. The bone marrow was collected using phosphate buffered saline (PBS). After collection, all samples were centrifuged, and the supernatant was discarded, leaving a small amount of PBS cell pellet. Smears were prepared on slides using the cell pellet. The slides were air-dried, fixed in 10% neutral formalin, and stained with 0.05% acridine orange.

### Statistical method

Statistical analysis was performed on the data of body weight, feed consumption, urine volume, hematology, clinical chemistry and organ weights using SAS program (version 9.4, SAS Institute Inc., USA). For the main groups and dosing period, the data was analyzed by Bartlett’s test for homogeneity of variance (significance level: 0.05). One-way analysis of variance (ANOVA) was employed on homogeneous data; then, if significant (significance level: 0.05), Dunnett’s test was applied for multiple comparisons (significance levels: 0.05 and 0.01, two-tailed). Kruskal–Wallis test was employed on heterogeneous data; then, if significant (significance level: 0.05), DSCF (Dwass–Steel–Critchlow–Fligner) was applied for multiple comparisons (significance levels: 0.05 and 0.01, two-tailed). For the recovery groups, the data was analyzed utilizing Folded F-test for homogeneity of variance (significance level: 0.05). Student’s t-test was employed on homogeneous data or Aspin-Welch t-test was employed on heterogeneous data for confirming significance (significance levels: 0.05 and 0.01, two-tailed).

## Results

### Acute oral toxicity

The acute oral toxicity of HemoHIM G was investigated in SD rats according to OECD Test guideline 423. In the preliminary test of this study, a single oral administration of the test substance at a dose limit of 5000 mg/10 mL/kg to one male and one female rat resulted in no deaths, general symptoms, or weight changes. Based on these findings, 5000 mg/kg of the test substance was orally administered once to five male and five female animals in each group. Throughout the observation period, no deaths occurred in the control, or 5000 mg/kg administration groups, and no significant changes in general symptoms or body weight were observed (data not shown). Upon completion of the observation period, no abnormal findings were noted on visual inspection during the autopsy (data not shown).

### 28-Day repeated dose oral toxicity

A repeated-dose oral toxicity study was conducted in accordance with the OECD Guideline 407 [17]. HemoHIM G was administered orally to SD rats (five rats/sex/group) at graduated dose levels of 0 mg/kg/day for control (G1), 1250 mg/kg/day for low dose (G2), 2500 mg/kg/day for mid-dose (G3), and 5000 mg/kg/day for high dose (G4). Vehicle or HemoHIM G was administered to each rat once daily for 28 consecutive days.

#### Mortality, clinical observations and gross pathology

All animals survived until the scheduled euthanasia. No item-related clinical signs were observed at any of the doses tested in either sex. Additionally, no item-related test findings were noted in gross pathology performed at the end of the treatment period.

#### Body weights and feed consumption

**Fig. 1:**
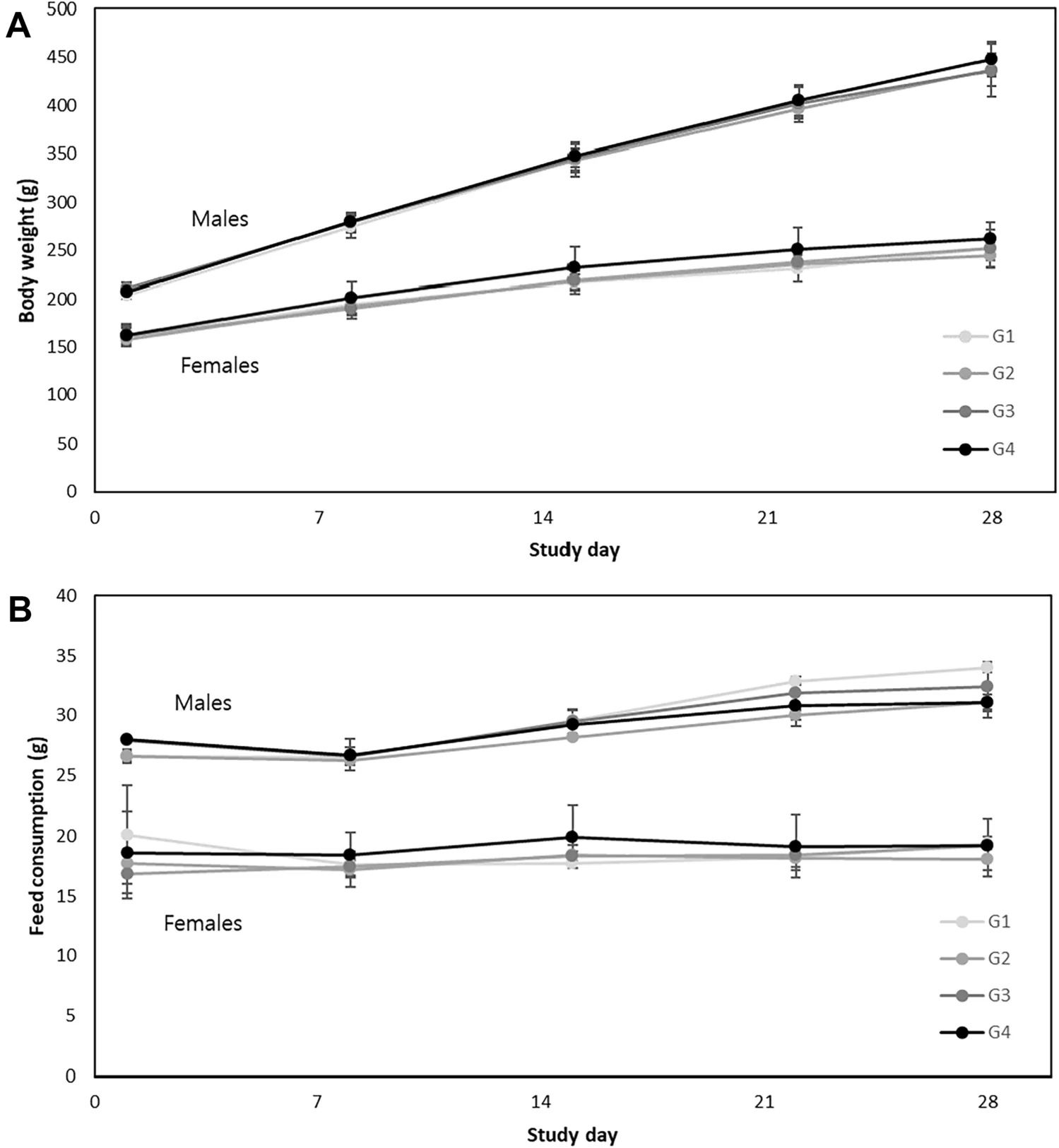
**A** Mean body weights and **B** Mean feed consumption in 28-day repeated dose oral toxicity. HemoHIM G was administered to rats at graduated dose levels: 0 mg/kg/day (G1), 1250 mg/kg/day (G2), 2500 mg/kg/day (G3), and 5000 mg/kg/day (G4)

No test substances related to changes in body weight or weight gain were observed in either sex at any of the doses tested throughout the observation period (Fig. 1A). Normal feed consumption was observed for both sexes at all tested doses during the observation period (Fig. 1B).

#### Hematology and clinical chemistry

At the end of the observation period, no adverse effects were observed in hematological or clinical chemistry parameters in either sex compared to their respective vehicle control groups. However, statistical significance was noted for the total erythrocyte count (RBC) and hematocrit value (Hct) in the female 2500 and 5000 mg/kg/day groups, although no associated clinical symptoms were observed. Additionally, there was a statistically significant difference in the A/G ratio between the male 1250 and 5000 mg/kg/day groups. However, a simple increase in the A/G ratio was not clinical significant [22], and no statistical significance was observed for total protein and albumin. Therefore, we concluded that the findings were not toxicologically significant (Tables 1, 2).

**Table 1:** Hematology values for male and female rats in 28-day repeated dose oral toxicity

	Males				Females			
	G1	G2	G3	G4	G1	G2	G3	G4
RBC (10^6^ cells/μL)	7.29 ± 0.22	7.26 ± 0.20	7.32 ± 0.32	7.15 ± 0.09	7.69 ± 0.27	7.42 ± 0.37	7.16 ± 0.28*	7.21 ± 0.19*
Hb (g/dL)	14.9 ± 0.1	15.0 ± 0.5	14.8 ± 0.5	14.6 ± 0.5	15.3 ± 00.3	15.0 ± 0.6	14.9 ± 0.8	14.6 ± 0.4
Hct (%)	42.9 ± 0.7	43.0 ± 1.1	42.2 ± 1.1	41.9 ± 1.1	43.7 ± 1.0	41.5 ± 1.4	41.6 ± 2.0	41.2 ± 0.8*
MCV (fL)	58.9 ± 1.0	59.2 ± 0.9	57.6 ± 1.9	585.5 ± 1.4	56.8 ± 0.9	56.0 ± 2.1	58.0 ± 1.1	57.1 ± 1.0
MCH (pg)	20.4 ± 0.5	20.7 ± 0.9	20.3 ± 0.8	20.4 ± 0.7	20.3 ± 0.5	20.2 ± 0.5	20.8 ± 0.7	20.3 ± 0.3
MCHC (g/dL)	34.7 ± 0.5	34.9 ± 0.6	35.1 ± 0.5	34.8 ± 0.5	35.8 ± 0.3	36.2 ± 0.5	35.9 ± 0.7	35.6 ± 0.6
Plt (10^3^ cells/μL)	916 ± 202	1000 ± 97	1047 ± 119	1025 ± 67	991 ± 95	1045 ± 113	1054 ± 68	1056 ± 80
WBC (10^3^ cells/μL)	11.29 ± 1.02	12.70 ± 2.18	11.09 ± 1.40	11.12 ± 1.98	7.98 ± 0.99	9.38 ± 1.46	8.27 ± 2.32	9.69 ± 2.45

Each value was presented as mean ± SD (*n* = 5). Significantly different from control by Dunnett’s t-test: *p < 0.05

**Table 2:** Clinical chemistry values for male and female rats in 28-day repeated dose oral toxicity

	Males				Females			
	G1	G2	G3	G4	G1	G2	G3	G4
ALT (U/L)	28.5 ± 3.0	25.1 ± 2.3	27.7 ± 2.7	27.0 ± 2.9	22.5 ± 3.6	20.9 ± 4.0	25.1 ± 3.5	23.4 ± 4.8
AST (U/L)	76.0 ± 3.5	72.0 ± 1.8	75.4 ± 6.0	75.7 ± 7.7	77.8 ± 9.6	73.5 ± 3.6	84.6 ± 17.1	78.3 ± 4.9
ALP (U/L)	464.5 ± 77.4	467.6 ± 84.1	399.6 ± 93.5	456.6 ± 75.2	343.5 ± 109.6	300.5 ± 85.4	365.9 ± 88.1	300.9 ± 52.2
Glu (mg/dL)	118 ± 3	123 ± 16	122 ± 16	113 ± 5	111 ± 8	114 ± 7	102 ± 6	107 ± 6
BUN (mg/dL)	12.8 ± 1.1	13.6 ± 1.8	12.2 ± 0.8	11.5 ± 0.7	15.9 ± 1.4	17.5 ± 3.6	16.1 ± 3.9	15.9 ± 3.0
Crea (mg/dL)	0.45 ± 0.03	0.42 ± 0.01	0.44 ± 0.02	0.42 ± 0.02	0.51 ± 0.06	0.52 ± 0.05	0.50 ± 0.02	0.50 ± 0.06
T.Chol (mg/dL)	76 ± 20	59 ± 19	74 ± 19	70 ± 8	74 ± 10	82 ± 13	71 ± 12	77 ± 24
Trig (mg/dL)	54 ± 19	45 ± 11	47 ± 12	55 ± 15	10 ± 3	18 ± 7	15 ± 9	11 ± 2
TP (g/dL)	5.5 ± 0.2	5.5 ± 0.2	5.6 ± 0.1	5.5 ± 0.2	5.9 ± 0.5	6.1 ± 0.2	5.7 ± 0.2	5.8 ± 0.2
ALB (g/dL)	2.3 ± 0.1	2.4 ± 0.1	2.4 ± 0.1	2.4 ± 0.1	2.6 ± 0.1	2.8 ± 0.2	2.7 ± 0.2	2.7 ± 0.1
A/G ratio	0.71 ± 0.03	0.76 ± 0.02*	0.75 ± 0.02	0.78 ± 0.04*	0.83 ± 0.09	0.85 ± 0.10	0.88 ± 0.06	0.84 ± 0.04

Each value was presented as mean ± SD (*n* = 5). Significantly different from control by Dunnett’s t-test: *p < 0.05

#### Organ weights and histopathology

No significant changes in organ weight, considered toxicologically significant, were observed in the males and females in the groups of 1250, 2500, and 5000 mg/kg/day. However, statistical significance was noted for the relative organ weights in the liver of the male high-dose group and the spleen of the female mid-dose group compared with the control group. During the autopsy, no morphological changes were observed in the organs, and no abnormal changes were observed in the hematology and blood chemical test results, which were deemed toxicological significance. Therefore, based on available evidence, a direct relationship with HemoHIM G could not be determined (Table 3).

**Table 3:** Relative organ weight (%) for male and female rats in 28-day repeated dose oral toxicity

	Males				Females			
	G1	G2	G3	G4	G1	G2	G3	G4
Fasting Body Weight (g)	413.9 ± 18.4	405.8 ± 14.3	409.0 ± 21.7	413.1 ± 17.6	232.2 ± 15.4	232.1 ± 6.8	235.2 ± 12.6	243.8 ± 19.9
Brain	0.49 ± 0.04	0.50 ± 0.04	0.49 ± 0.03	0.49 ± 0.03	0.83 ± 0.04	0.81 ± 0.04	0.80 ± 0.06	0.79 ± 0.05
Heart	0.33 ± 0.02	0.33 ± 0.03	0.33 ± 0.01	0.33 ± 0.01	0.36 ± 0.02	0.38 ± 0.02	0.37 ± 0.02	0.36 ± 0.03
Kidneys	0.70 ± 0.07	0.75 ± 0.03	0.73 ± 0.03	0.77 ± 0.03	0.77 ± 0.04	0.79 ± 0.05	0.72 ± 0.03	0.76 ± 0.06
Liver	3.00 ± 0.16	3.05 ± 0.29	3.24 ± 0.16	3.41 ± 0.14*	2.99 ± 0.14	3.07 ± 0.15	3.03 ± 0.15	3.14 ± 0.20
Spleen	0.20 ± 0.03	0.22 ± 0.04	0.21 ± 0.03	0.24 ± 0.04	0.22 ± 0.03	0.23 ± 0.02	0.23 ± 0.02*	0.25 ± 0.02

Each value was presented as mean ± SD (*n* = 5). Significantly different from control by Dunnett’s t-test: *p < 0.05

### 13-week repeated dose oral toxicity

A repeated-dose oral toxicity study was conducted in accordance with OECD Guideline 408 [18]. HemoHIM G was orally administered to Sprague–Dawley rats (10 rats/sex/group) at graduated dose levels of 0 mg/kg/day for control (G1) and control recovery groups (G1R), 1250 mg/kg/day for low dose (G2), 2500 mg/kg/day for mid-dose (G3), and 5000 mg/kg/day for high dose (G4) and high dose recovery groups (G4R) for 13 consecutive weeks. Vehicle or HemoHIM G was administered to each rat once daily for 13 consecutive weeks. During the observation period, clinical and detailed clinical signs, measurement of body weight and feed consumption, ophthalmological examinations, and urinalysis were observed. At the end of the observation period, hematological and clinical chemistry examinations, observation of the estrus cycle, organ weight measurements, gross postmortem examinations, and histopathological examinations were performed.

#### Mortality, clinical observations and gross pathology

No deaths or abnormal clinical signs were observed during the dosing period in either the control or the HemoHIM G-treated groups, irrespective of sex. A detailed examination of the clinical signs revealed no abnormal changes in the control or HemoHIM G-treated groups. Ophthalmological examination did not reveal any abnormalities in any animal (data not shown). Additionally, regarding the results of observation of the estrus cycle, compared to the histopathological examination of the female genital organs, there were no significant changes (data not shown). There were also no treatment related changes was observed following the recovery periods.

#### Body weights and feed consumption

**Fig. 2:**
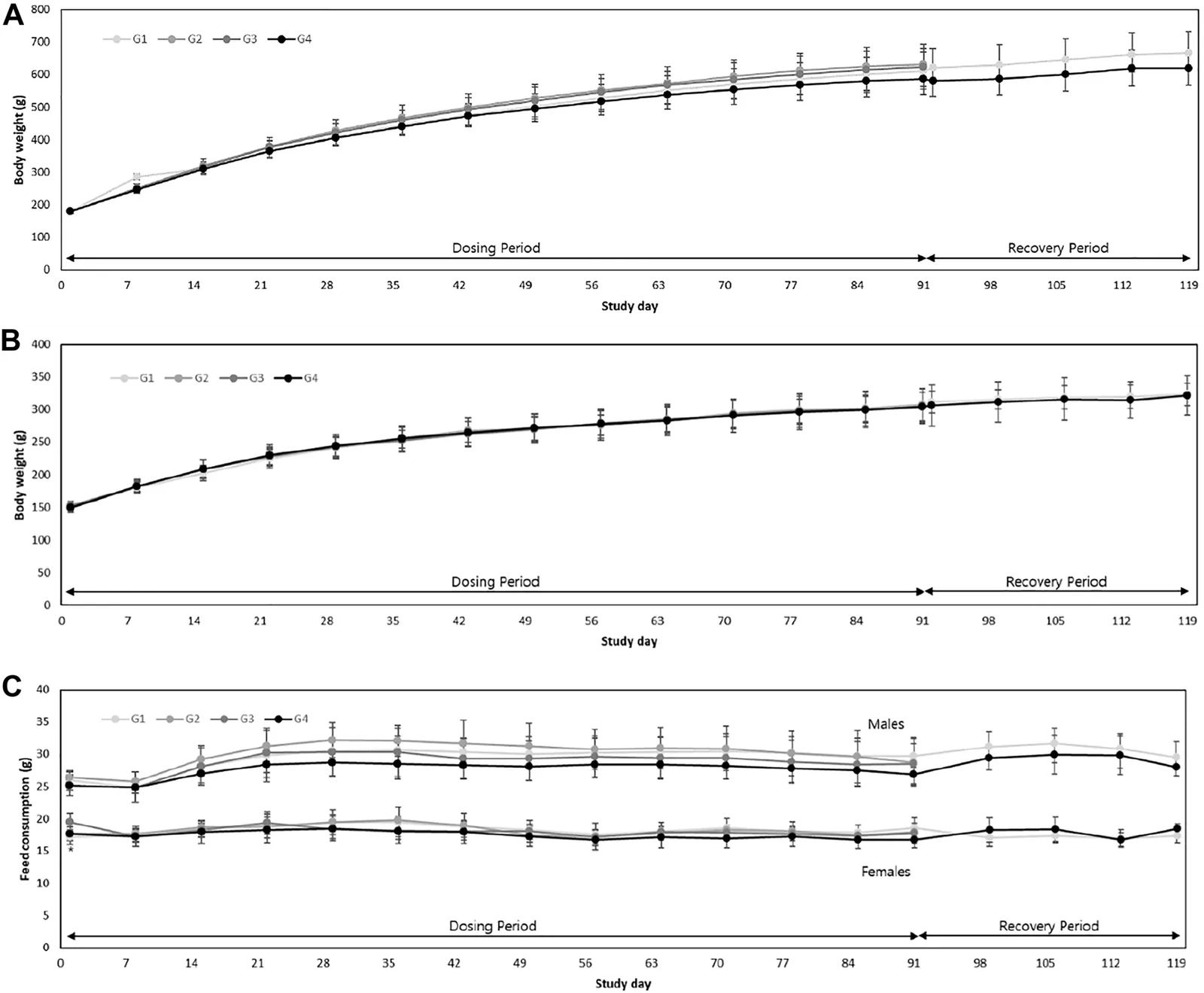
Mean body weights in **A** male and **B** female SD rats, and **C** mean feed consumption in 13-week repeated dose oral toxicity. HemoHIM G was administered to rats at graduated dose levels, including a control group (G1) receiving 0 mg/kg/day, and treatment groups (G2-4) receiving doses of 1250, 2500, 5000 mg/kg/day, respectively

Throughout the dosing period, no significant toxicological changes in body weight or feed consumption were observed in HemoHIM G-treated groups of either sex. However, in the male high-dose group, the observed differences were consistent with changes in feed consumption due to individual variations. Furthermore, the differences, including those with statistical significance, were not considered to be effects related to the test substance because they sporadically occurred within the normal variability commonly observed in animals (Fig. 2). There were also no treatment related changes was observed following the recovery periods.

#### Hematology, clinical chemistry and urinalysis

Administration of HemoHIM G did not result in significant toxicological changes in hematological and clinical chemistry parameters in either sex. However, certain changes, such as a decrease in RBC count, an increase and decrease in RBC-related parameters (Hb, Hemoglobin concentration; Hct; MCV, mean corpuscular hemoglobin; and MCH, mean corpuscular hemoglobin), and an increase in reticulocytes (Rec), were observed and considered to be related to the test substance. These changes were dose-dependent and tended to resolve after recovery. Similarly, bilirubin and ketone bodies in the urine tended to increase, but no significant toxic effects were determined based on clinical and histopathological examinations. Therefore, the evidence was insufficient to establish a toxic effect (Table 4, 5). There were also no treatment related changes was observed following the recovery periods. In urinalysis, bilirubin and ketone bodies were found in the urine of both control and HemoHIM G-treated groups, with slightly higher levels in the treated group. Changes in Ketone bodies and Bilirubin were related to the treatment but not indicative of kidney or liver damage. Other parameter differences were minor and not related to treatment (data not shown). Overall, there was insufficient evidence of toxic effects.

**Table 4:** Hematology values for male and female rats in 13-week repeated dose oral toxicity

	Males				Females			
	G1	G2	G3	G4	G1	G2	G3	G4
RBC (10^6^ cells/μL)	8.11 ± 0.36	8.18 ± 0.36	7.64 ± 0.20**	7.62 ± 0.36**	7.53 ± 0.26	7.03 ± 0.28**	6.95 ± 0.42**	6.71 ± 0.33**
Hb (g/dL)	14.6 ± 0.7	14.7 ± 0.5	14.4 ± 0.3	14.1 ± 0.5	14.3 ± 0.5	13.8 ± 0.7	13.5 ± 0.5**	13.5 ± 0.3**
Hct (%)	41.6 ± 1.8	41.4 ± 1.4	40.7 ± 0.9	39.9 ± 1.4*	40.1 ± 1.3	38.5 ± 1.7*	37.6 ± 1.4**	37.6 ± 0.7**
MCV (fL)	51.4 ± 1.6	50.7 ± 1.3	53.3 ± 1.7*	52.5 ± 2.1	53.2 ± 1.4	54.7 ± 1.2	54.2 ± 1.8	56.1 ± 2.7**
MCH (pg)	18.0 ± 0.7	17.9 ± 0.6	18.8 ± 0.6*	18.8 ± 0.8	19.0 ± 0.5	19.7 ± 0.5	19.5 ± 0.7	20.2 ± 0.7**
MCHC (g/dL)	35.0 ± 0.5	35.4 ± 0.6	35.3 ± 0.4	35.3 ± 0.5	35.7 ± 0.4	35.9 ± 0.2	35.9 ± 0.4	36.0 ± 0.7
Plt (10^3^ cells/μL)	1028 ± 117	992 ± 1	1037 ± 171	1066 ± 86	914 ± 78	993 ± 77	929 ± 156	939 ± 78
Reti (%)	3.27 ± 0.56	3.64 ± 0.47	4.48 ± 0.38**	5.34 ± 0.59**	2.75 ± 0.40	3.42 ± 0.47*	3.97 ± 0.45**	5.05 ± 0.64**
WBC (10^3^ cells/μL)	10.04 ± 2.05	10.03 ± 3.43	11.25 ± 1.21	10.86 ± 3.30	4.43 ± 1.62	3.65 ± 1.24	3.15 ± 0.95	4.39 ± 1.37
Neu (%)	16.1 ± 5.1	20.1 ± 7.0	14.2 ± 4.8	19.2 ± 4.1	14.9 ± 4.9	15.0 ± 4.4	18.2 ± 4.8	18.9 ± 5.0
Lym (%)	74.9 ± 5.7	69.6 ± 6.8	77.1 ± 5.8	70.9 ± 4.6	75.2 ± 5.2	76.5 ± 5.2	72.3 ± 4.3	73.3 ± 4.4
Mono (%)	7.8 ± 1.6	8.8 ± 1.2	7.5 ± 1.5	8.8 ± 1.9	7.9 ± 1.1	6.6 ± 1.5	7.6 ± 1.6	6.4 ± 1.1*
Eoso (%)	1.0 ± 0.2	1.2 ± 0.4	1.0 ± 0.3	0.8 ± 0.2	1.8 ± 0.6	1.6 ± 0.5	1.7 ± 0.4	1.2 ± 0.6
Baso (%)	0.2 ± 0.1	0.3 ± 0.1	0.3 ± 0.1	0.3 ± 0.1	0.3 ± 0.1	0.3 ± 0.1	0.4 ± 0.2	0.3 ± 0.1
PT (Sec)	18.7 ± 0.6	18.1 ± 0.8	18.6 ± 0.7	18.9 ± 1.0	17.9 ± 0.6	1.81 ± 1.1	17.7 ± 0.7	18.7 ± 1.0
APTT (Sec)	14.1 ± 1.2	13.2 ± 1.4	13.6 ± 2.0	13.8 ± 2.2	14.1 ± 2.0	14.4 ± 2.2	13.9 ± 1.8	13.9 ± 2.4

Each value was presented as mean ± SD. Significantly different from control by Dunnett’s t-test: *p < 0.05, **p < 0.01

**Table 5:** Clinical chemistry values for male and female rats in 13-week repeated dose oral toxicity

	Males				Females			
	G1	G2	G3	G4	G1	G2	G3	G4
ALT (U/L)	28.0 ± 2.6	27.3 ± 3.1	23.5 ± 3.3**	23.3 ± 3.2**	27.9 ± 9.9	23.4 ± 5.2	25.8 ± 7.4	19.1 ± 2.6*
AST (U/L)	93.2 ± 16.9	82.9 ± 17.1	75.7 ± 13.1	77.6 ± 22.1	96.2 ± 25.3	81.7 ± 17.4	86.6 ± 18.3	72.9 ± 14.7
ALP (U/L)	225.9 ± 36.6	256.1 ± 33.8	244.2 ± 48.6	235.4 ± 61.4	121.2 ± 19.5	118.4 ± 18.9	136.6 ± 38.4	140.9 ± 48.6
GGT (U/L)	0.08 ± 0.09	0.03 ± 0.09	0.01 ± 0.01	0.05 ± 0.08	0.55 ± 0.22	0.60 ± 0.29	0.58 ± 0.26	0.60 ± 0.25
Glu (mg/dL)	158 ± 16	166 ± 13	156 ± 17	154 ± 18	137 ± 21	145 ± 24	129 ± 16	124 ± 12
BUN (mg/dL)	13.0 ± 1.3	14.1 ± 1.4	13.9 ± 1.5	13.1 ± 1.0	18.3 ± 2.9	17.4 ± 2.8	17.1 ± 2.2	16.5 ± 2.7
Crea (mg/dL)	0.46 ± 0.04	0.47 ± 0.05	0.46 ± 0.05	0.45 ± 0.04	0.66 ± 0.06	0.61 ± 0.07	0.61 ± 0.04	0.60 ± 0.09
T.Bili (mg/dL)	0.06 ± 0.03	0.07 ± 0.02	0.08 ± 0.02	0.08 ± 0.02	0.07 ± 0.03	0.08 ± 0.04	0.09 ± 0.03	0.10 ± 0.03
T.Chol (mg/dL)	71 ± 13	75 ± 16	73 ± 20	61 ± 1	89 ± 16	82 ± 19	87 ± 16	76 ± 19
Trig (mg/dL)	41 ± 15	62 ± 33	71 ± 37	57 ± 17	18 ± 6	25 ± 17	18 ± 6	18 ± 8
TP (g/dL)	5.8 ± 0.2	5.9 ± 0.3	5.9 ± 0.3	5.9 ± 0.3	6.7 ± 0.4	6.7 ± 0.3	6.7 ± 0.4	6.3 ± 0.3
ALB (g/dL)	2.3 ± 0.1	2.4 ± 0.2	2.4 ± 0.1	2.4 ± 0.1	3.0 ± 0.3	3.2 ± 0.2	3.1 ± 0.3	2.9 ± 0.2
A/G ratio	0.65 ± 0.04	0.68 ± 0.04	0.67 ± 0.06	0.70 ± 0.04	0.82 ± 0.06	0.88 ± 0.06	0.85 ± 0.05	0.87 ± 0.07
Phos (mg/dL)	6.30 ± 0.48	6.34 ± 0.18	6.44 ± 0.42	6.56 ± 0.52	4.86 ± 0.77	4.98 ± 1.00	4.62 ± 0.60	5.12 ± 0.63
Ca (mg/dL)	9.2 ± 0.2	9.3 ± 0.3	9.4 ± 0.3	9.2 ± 0.2	9.5 ± 0.2	9.6 ± 0.3	9.6 ± 0.3	9.2 ± 0.2
Na (mmol/L)	141.4 ± 1.2	141.1 ± 0.7	141.1 ± 0.9	140.3 ± 0.8	140.5 ± 1.1	140.6 ± 0.9	140.3 ± 1.0	139.9 ± 1.1
K (mmol/L)	4.75 ± 0.29	4.57 ± 0.20	4.60 ± 0.20	4.75 ± 0.32	4.03 ± 0.33	4.21 ± 0.21	4.02 ± 0.25	4.14 ± 0.18
Cl (mmol/L)	103.0 ± 1.3	102.4 ± 1.4	101.9 ± 1.3	101.5 ± 1.0	104.1 ± 1.8	103.6 ± 1.3	103.5 ± 1.2	102.9 ± 1.8
TBA (mmol/L)	15.2 ± 7.1	14.8 ± 8.6	16.5 ± 10.8	17.5 ± 10.3	11.2 ± 6.7	10.4 ± 1.7	10.9 ± 3.8	12.4 ± 5.0
Urea (mg/dL)	28 ± 3	30 ± 3	30 ± 3	28 ± 2	39 ± 6	37 ± 6	37 ± 5	35 ± 6
HDL (mg/dL)	20.3 ± 2.4	22.1 ± 4.3	21.0 ± 4.1	19.8 ± 2.4	27.3 ± 4.0	26.1 ± 5.0	27.1 ± 4.5	25.3 ± 5.1
LDL (mg/dL)	4.4 ± 1.4	3.6 ± 0.7	4.6 ± 1.7	3.4 ± 0.9	3.3 ± 0.4	2.8 ± 0.6	3.2 ± 0.5	2.8 ± 1.0
T4 (ng/dL)	64.9 ± 5.9	66.2 ± 8.3	66.8 ± 13.5	60.0 ± 14.8	32.5 ± 6.9	32.6 ± 8.0	28.4 ± 7.4	29.9 ± 10.2
T3 (ng/dL)	0.808 ± 0.157	0.770 ± 0.090	0.853 ± 0.147	0.805 ± 0.094	0.640 ± 0.170	0.637 ± 0.120	0.528 ± 0.082	0.570 ± 0.106
TSH (ng/dL)	1.795 ± 0.609	2.490 ± 1.395	2.271 ± 1.187	2.699 ± 1.664	3.433 ± 2.534	4.429 ± 2.265	4.774 ± 2.371	5.315 ± 3.797

Each value was presented as mean ± SD. Significantly different from control by Dunnett’s t-test: *p < 0.05, **p < 0.01

#### Organ weights and histopathology

An increase and a tendency of increased spleen weight were observed in the HemoHIM G-treated main groups compared to the controls. Considering the results of hematological and histopathological examinations of the spleen, these changes were determined to be related to the test substance. However, no such changes were observed in the recovery group. Notably, these differences were not considered toxicologically significant, as the mean values fell within the normal range typically observed in animals (Table 6, 7). There were also no treatment related changes was observed following the recovery periods. Based on the conditions of this study, the test substance-related death and toxicologically significant changes were not observed in the HemoHIM G-treated groups. Therefore, the NOAEL (No Observable Adverse Effect Level) of the test substance, HemoHIM G, was considered to be at 5000 mg/kg/day for both sexes of SD rats.

**Table 6:** Relative organ weight (%) for male rats in 13-week repeated dose oral toxicity

	G1	G2	G3	G4
Fasting body weight (g)	582.1 ± 56.5	606.2 ± 45.3	596.7 ± 66.8	566.7 ± 51.6
Adrenal glands	0.0108 ± 0.0014	0.0098 ± 0.0018	0.0095 ± 0.0014	0.0105 ± 0.0015
Brain	0.3821 ± 0.0301	0.3717 ± 0.0221	0.3599 ± 0.0423	0.3793 ± 0.0209
Epididymis	0.2782 ± 0.0388	0.2769 ± 0.0393	0.2719 ± 0.0304	0.2705 ± 0.0272
Heart	0.717 ± 0.0194	0.2626 ± 0.0130	0.2752 ± 0.00178	0.2814 ± 0.0225
Kidneys	0.5831 ± 0.0416	0.5553 ± 0.0443	0.5961 ± 0.0501	0.6065 ± 0.0457
Liver	2.5691 ± 0.1844	2.5207 ± 0.1713	2.6497 ± 0.1803	2.7786 ± 0.2267
Pituitary gland	0.0026 ± 0.0003	0.0023 ± 0.0003	0.0026 ± 0.0004	0.0027 ± 0.0004
SV-CG and Prostate Gland	0.6856 ± 0.0926	0.6624 ± 0.0777	0.6764 ± 0.1137	0.6670 ± 0.0937
Spleen	0.1754 ± 0.0248	0.1669 ± 0.0121	0.1747 ± 0.0187	0.1922 ± 0.0262
Testis	0.6521 ± 0.0684	0.6269 ± 0.0746	0.6037 ± 0.0689	0.6426 ± 0.0890
Thymus	0.0563 ± 0.0123	0.0609 ± 0.0104	0.0601 ± 0.0115	0.0618 ± 0.0108
Thyroid gland with parathyroid gland	0.0063 ± 0.0011	0.0059 ± 0.0007	0.0059 ± 0.0010	0.0065 ± 0.0010

Each value was presented as mean ± SD

**Table 7:** Relative organ weight (%) for female rats in 13-week repeated dose oral toxicity

	G1	G2	G3	G4
Fasting body weight (g)	288.2 ± 27.9	289.2 ± 22.8	288.3 ± 19.8	283.2 ± 26.4
Adrenal glands	0.0229 ± 0.0031	0.0225 ± 0.0046	0.0242 ± 0.0030	0.0224 ± 0.0039
Brain	0.6846 ± 0.0646	0.7034 ± 0.0566	0.6923 ± 0.0474	0.6927 ± 0.0604
Heart	0.3241 ± 0.0307	0.3190 ± 0.0286	0.3161 ± 0.0232	0.3356 ± 0.0206
Kidneys	0.6187 ± 0.0428	0.6400 ± 0.0505	0.6380 ± 0.0528	0.6933 ± 0.0532**
Liver	2.5900 ± 0.1768	2.5776 ± 0.1466	2.6400 ± 0.1523	2.7115 ± 0.2120
Ovaries	0.0311 ± 0.0055	0.0328 ± 0.0047	0.0302 ± 0.0045	0.0302 ± 0.0039
Pituitary gland	0.0070 ± 0.0013	0.0065 ± 0.0010	0.0066 ± 0.0012	0.0065 ± 0.0012
Spleen	0.1894 ± 0.0237	0.1893 ± 0.0179	0.1900 ± 0.0110	0.2273 ± 0.0289**
Thymus	0.0937 ± 0.0244	0.0969 ± 0.0113	0.0922 ± 0.0233	0.1155 ± 0.0208
Thyroid gland with parathyroid gland	0.0094 ± 0.0011	0.0095 ± 0.0009	0.0087 ± 0.0012	0.0099 ± 0.0016
Uterus with CrV	0.2388 ± 0.0544	0.3053 ± 0.0847	0.2502 ± 0.0757	0.2284 ± 0.0494

Each value was presented as mean ± SD (*n* = 5). Significantly different from control by Dunnett’s t-test: **p < 0.01

### Bacterial reverse mutation test

An in vitro bacterial reverse mutation assay was conducted using HemoHIM G in accordance with OECD Guideline 471. No precipitation and cytotoxicity in the form of background lawn reduction and the revertant count were observed at all the concentrations tested (ranging from 313 to 5000 μg HemoHIM G/plate both in the presence (S9) and absence of metabolic activation system when compared to vehicle control plates. The positive controls responded as expected. The mean number of revertant colonies was within the acceptable range of historical data for the vehicle and positive controls (Table 8).

**Table 8:** Mean number of revertants ± SD in the absence or presence of metabolic activation system in bacterial reverse mutation test

Strain	Test substance	Dose (μg/plate)	S9 mix ( −)		S9 mix ( +)	
			1st	2nd	1st	2nd
TA98	HemoHIM G	0	22 ± 3	16 ± 1	30 ± 2	29 ± 2
		313	21 ± 1	15 ± 3	28 ± 2	30 ± 3
		625	20 ± 2	18 ± 2	28 ± 1	34 ± 3
		1250	19 ± 2	19 ± 5	35 ± 2	31 ± 2
		2500	23 ± 3	19 ± 3	34 ± 2	31 ± 4
		5000	24 ± 2	20 ± 1	34 ± 1	31 ± 3
	2-Nitrofluorene	5.0	646 ± 19	626 ± 11	–	–
	2-aminoanthracene	1.0	–	–	401 ± 18	270 ± 5
TA100	HemoHIM G	0	123 ± 5	106 ± 7	133 ± 4	120 ± 5
		313	128 ± 2	109 ± 8	122 ± 6	118 ± 2
		625	123 ± 3	108 ± 6	134 ± 4	120 ± 4
		1250	131 ± 2	116 ± 6	139 ± 2	119 ±
		2500	141 ± 4	113 ± 7	131 ± 1	113 ± 5
		5000	141 ± 2	126 ± 2	144 ± 5	129 ± 9
	Sodium azide	1.5	785 ± 18	781 ± 5	–	–
	2-aminoanthracene	2.0	–	–	867 ± 31	918 ± 104
TA1535	HemoHIM G	0	14 ± 2	15 ± 2	13 ± 2	16 ± 2
		313	12 ± 1	16 ± 2	11 ± 1	15 ± 2
		625	16 ± 1	15 ± 2	16 ± 2	15 ± 2
		1250	16 ± 1	15 ± 3	14 ± 1	14 ± 1
		2500	17 ± 1	15 ± 4	15 ± 1	18 ± 1
		5000	16 ± 1	17 ± 3	16 ± 2	17 ± 2
	Sodium azide	1.5	624 ± 18	568 ± 15	–	–
	2-aminoanthracene	3.0	–	–	155 ± 41	185 ± 6
TA1537	HemoHIM G	0	9 ± 1	9 ± 2	18 ± 1	16 ± 2
		313	10 ± 2	9 ± 2	17 ± 1	15 ± 2
		625	9 ± 1	9 ± 1	18 ± 2	15 ± 2
		1250	10 ± 2	9 ± 2	21 ± 1	14 ± 1
		2500	12 ± 1	9 ± 1	20 ± 1	18 ± 1
		5000	13 ± 2	11 ± 2	22 ± 2	17 ± 2
	9-Aminoacridine	80.0	551 ± 42	534 ± 11	–	–
	2-aminoanthracene	3.0	–	–	176 ± 7	185 ± 6
WP2*uvrA*	HemoHIM G	0	35 ± 4	36 ± 2	37 ± 2	34 ± 2
		313	34 ± 1	35 ± 2	39 ± 2	34 ± 1
		625	34 ± 4	37 ± 1	39 ± 2	35 ± 3
		1250	39 ± 1	36 ± 3	37 ± 2	35 ± 5
		2500	38 ± 3	36 ± 1	37 ± 2	34 ± 2
		5000	40 ± 2	39 ± 2	41 ± 2	37 ± 4
	4-Nitroquinoline N-oxide	0.3	808 ± 46	824 ± 96	–	–
	2-aminoanthracene	10.0	–	–	514 ± 38	570 ± 16

Mean number of revertants ± SD in the absence or presence of metabolic activation system

### In vitro mammalian chromosomal aberration test

An in vitro mammalian chromosomal aberration assay was conducted using HemoHIM G following OECD Guideline 473. In the main study, the frequency of cells with chromosomal aberrations in the short-term treatments with and without metabolic activation, as well as in the continuous treatment without metabolic activation, did not show statistically significant differences compared with the negative control group. However, the positive controls, mitomycin C and benzo[a]pyrene, showed statistically significant increases in the proportion of cells with structural chromosomal aberrations. The results for the vehicle and positive controls were as expected, confirming the sensitivity of the test system, the effectiveness of the S9 mix, and the validity of the assay (Table 9).

**Table 9:** Chromosome aberration assay in in vitro mammalian chromosomal aberration test

Test substance	Dose (μg/plate)	RPD (%)	PD	S9 mix	Trt-Rec Time (hr)	Cell analyzed	Structural aberrations									Numerical aberrations			Others^a^
							Ctb	Csb	Cte	Cse	Frg	Gap		Total (%)		End	Pol	Total (%)	
												Ctg	Csg	Gap ( −)	Gap ( +)				
HemoHIM G	0	100	1.52	–	6–18	150	0	0	0	0	0	0	0	1 (0.3)	1 (0.3)	0	0	0 (0.0)	0
							1	0	0	0	0	0	0			0	0		
	78	96.0	–	–	6–18	Not observed													
	156	95.4	–	–	6–18	150	0	0	0	0	0	0	0	0 (0.0)	0 (0.0)	0	0	0 (0.0)	0
							0	0	0	0	0	0	0			0	0		
	313	89.6	–	–	6–18	150	0	0	0	0	0	0	0	0 (0.0)	1 (0.3)	0	0	0 (0.0)	0
							0	0	0	0	0	1	0			0	0		
	625	86.8	–	–	6–18	150	0	0	0	0	0	0	0	0 (0.0)	0 (0.0)	0	0	0 (0.0)	0
							0	0	0	0	0	0	0			0	0		
MMC	0.1	49.6	–	–	6–18	150	9	0	18	1	0	0	0	53** (17.7)	53 (17.7)	0	0	0 (0.0)	0
							9	0	15	1	0	0	0			0	0		
HemoHIM G	0	100	1.53	+	6–18	150	0	0	0	0	0	0	0	1 (0.3)	2 (0.7)	0	0	0 (0.0)	0
							1	0	0	0	0	1	0			0	0		
	78	96.0	–	+	6–18	Not observed													
	156	92.9	–	+	6–18	150	0	0	0	0	0	0	0	0 (0.0)	0 (0.0)	0	0	0 (0.0)	0
							0	0	0	0	0	0	0			0	0		
	313	90.2	–	+	6–18	150	0	0	0	0	0	0	0	1 (0.3)	1 (0.3)	0	0	0 (0.0)	0
							1	0	0	0	0	0	0			0	0		
	625	87.4	–	+	6–18	150	0	0	0	0	0	0	0	0 (0.0)	0 (0.0)	0	0	0 (0.0)	0
							0	0	0	0	0	0	0			0	0		
B[a]P	20	46.0	–	+	6–18	150	10	0	18	0	0	0	0	54** (18.0)	54 (18.0)	0	0	0 (0.0)	0
							12	0	16	0	0	2	0			0	0		
HemoHIM G	0	100	1.53	–	24–0	150	0	0	0	0	0	0	0	0 (0.0)	0 (0.0)	0	0	0 (0.0)	0
							0	0	0	0	0	0	0			0	0		
	78	96.0	–	–	24–0	Not observed													
	156	94.5	–	–	24–0	150	0	0	0	0	0	0	0	0 (0.0)	0 (0.0)	0	0	0 (0.0)	0
							0	0	0	0	0	0	0			0	0		
	313	89.8	–	–	24–0	150	0	0	0	0	0	0	0	0 (0.0)	0 (0.0)	0	0	0 (0.0)	0
							0	0	0	0	0	0	0			0	0		
	625	88.7	–	–	24–0	150	0	0	0	0	0	0	0	0 (0.0)	1 (0.3)	0	0	0 (0.0)	0
							0	0	0	0	0	1	0			0	0		
MMC	0.1	46.6	–	–	24–0	150	9	0	18	1	0	0	0	56** (18.7)	57 (19.0)	0	0	0 (0.0)	0
							10	0	17	1	0	1	0			0	0		

^a^Others were excluded from the number of cells with chromosomal aberrations. Significant difference from negative control by Fisher’s exact test: **p < 0.01

### Mammalian bone marrow erythrocyte micronucleus test

An in vivo mammalian bone marrow erythrocyte micronucleus assay was conducted in accordance with OECD Guideline 474. In the main study, there were no statistically significant differences in the incidence of micronucleated polychromatic erythrocytes (MNPCE) or the ratio of polychromatic erythrocytes (PCE) to total erythrocytes between the test substance groups and the negative control group. However, the positive control group showed a statistically significant increase in the incidence of MNPCE compared to the negative control group. The ratio of PCE to total erythrocytes in the positive control group was not significantly different from that in the negative control group (Table 10).

**Table 10:** Results of main study in male SD rats in mammalian bone marrow erythrocyte micronucleus test

Group		Dose (mg/kg/day)	Route	Hours after dosing	*N* = 5	PCE/(PCE + NCE)	MNPCE/PCE
Negative control	Water	0	P.O	24	Total	789/2500	7/20,000
					% (Mean ± SD)	31.6 ± 1.24	0.035 ± 0.022
Test substance	HemoHIM G	1250	P.O	24	Total	808/2500	6/20,000
					% (Mean ± SD)	32.3 ± 0.94	0.030 ± 0.021
		2500	P.O	24	Total	780/2500	6/20,000
					% (Mean ± SD)	31.2 ± 1.12	0.030 ± 0.021
		5000	P.O	24	Total	790/2500	8/20,000
					% (Mean ± SD)	31.6 ± 0.58	0.040 ± 0.029
Positive control	CPA	20	P.O	24	Total	798/2500	864^++^/20,000
					% (Mean ± SD)	31.9 ± 1.99	4.320 ± 0.202

*PCE* Polychromatic erythrocyte, *MNPCE* Micronucelated polychromatic erythrocyte, *CPA* Cyclophosphamid. Significant difference from negative control by Mann–Whitney *U-*test: ^++^*p* < 0.01

## Discussion

Ensuring the safety of functional foods is crucial as these products offer additional health advantages beyond fundamental nutrition to general consumers. Numerous countries have established regulatory measures to improve the safety of functional foods. They have mandated guidelines and regulations to guarantee the safety and quality of functional foods available in the market. For instance, in Korea, the approval process for functional foods necessitates submitting safety information, including the justification for dietary consumption, active ingredients or associated substances, assessment of daily intake, nutritional evaluation, biological benefits, human test data, and toxicity test data.

When evaluating the safety of functional foods, it is essential to rely on credible intake assessment data from reputable sources such as the National Health and Nutrition Examination Survey. To ensure a comprehensive safety evaluation, various indicators, such as hematological/biochemical tests, urine tests, vital signs, and body measurements, should be included in general toxicity tests. Toxicity assessments should adhere to the Test Guidelines established by the OECD and encompass tests such as acute toxicity, repeated dose toxicity (preferably conducted for 90 days), and genotoxicity. In addition, documented cases of adverse reactions have been presented. To substantiate functional health benefits, it is necessary to provide supporting evidence from in vitro and in vivo studies that elucidate the mechanism of action at the cellular or organismal level [23].

Oriental medicine employs Samul-tang to treat blood-related conditions like anemia, utilizing components like *Angelicae gigantis* radix, *Cnidii rhizoma*, *Paeoniae* radix, and *Rehmanniae* radix *preparata* [24, 25]. Previous studies have indicated that Samul-tang influences cellular processes in the bone marrow, hematopoietic stem cells, and blood cells (such as erythrocytes, leukocytes, and thrombocytes), along with key hematopoietic factors such as erythropoietin, granulocyte-colony stimulating factor (G-CSF), interleukins, and interferon-gamma [26, 27]. HemoHIM G is a mixed extract of *Angelica sinensis*, *Ligusticum chuanxiong*, and *Paeonia lactiflora*, with *Rehmannia* radix excluded from the four herbs found in Samul-tang. This composition raises expectations for HemoHIM G to show a range of activities inherent to herbal extracts, including antioxidant, neuroprotective, anti-inflammatory, and antinociceptive effects. This suggests its potential for adaptogenic properties comparable to those of Samul-tang. Nevertheless, available evidence regarding the toxicity of HemoHIM G remains limited.

Yang et al. investigated the toxicity of roots of *A. sinensis* injection using a chick embryo chorioallantoic membrane model [28]. The results showed that the injection did not inhibit the survival of chick embryos. There were no significant differences between the treatment groups and the negative control, suggesting that the toxicity of *A. sinensis* was very limited. The essential oil of *Ligusticum chuanxiong* contains compounds such as ligustilide (67.46%) and butylidenephthalide (5.06%) [29]. Ligustilide has anti-inflammatory and anti-apoptotic properties [30–32] but higher doses may be toxic to nerve cells [33]. The ligustilide in essential oils is usually stable [34]; therefore, its safety may reflect some of its properties. Additionally, the benzoic acid found in Paeonia is considered safe by the FDA as an antifungal agent in food [35]. Peony is generally well tolerated, with occasional gastrointestinal upset and allergic skin reactions, particularly after topical application. Dietary supplements containing peony do not require extensive Food and FDA approval in the United States [36]. However, high concentrations of certain constituents, such as phenol and pyrethrin I, can cause toxicity [37, 38], raising the need for a safety assessment of HemoHIM G.

This study aimed to investigate the acute and repeated-dose oral toxicity and genotoxicity of HemoHIM G, following the OECD Test guidelines. The bacterial reverse mutation test results indicated that HemoHIM G did not induce point mutations in bacterial tester strains, both in the presence and absence of metabolic activation, up to a concentration of 5000 μg/plate. Therefore, it was considered nonmutagenic. In the chromosome aberration assay conducted using cultured CHL/IU cells, HemoHIM G did not induce structural chromosome aberrations when treated up to a concentration of 625 μg/mL, under short-term exposure conditions with or without metabolic activation, as well as continuous exposure conditions without metabolic activation. Consequently, it was deemed non-clastogenic. Furthermore, the in vivo micronucleus test revealed that HemoHIM G did not induce micronuclei formation in the bone marrow polychromatic erythrocytes of male rats treated with up to 5000 mg/kg. Based on these findings, we concluded that HemoHIM G does not exhibit genotoxic potential.

The acute oral LD50 value of HemoHIM G was greater than 5000 mg/kg in male and female SD rats. However, in a 13-week repeated oral dose toxicity study, substance-related histopathological changes were observed in the spleen and bone marrow (sternum). Increased hematopoietic cellularity in the spleen and bone marrow was predominantly occupied by erythroid lineage cells, and the incidence of this change increased dose-dependently. Based on the incidence and correlation among the pathological results, changes in the spleen and bone marrow were considered to be related to the test substance. These changes were considered to affect the weight of the spleen, hematological changes, and urinalysis results. However, because the hematological parameters in the clinical pathology were within the normal range and were deemed to suggest an adequate compensatory response, it was considered not toxicological significance.

In particular, an increased number of macrophages containing brown granular pigments were observed in the red and white pulps of the spleen. Given that hemosiderin is a breakdown product of RBC and an increase in the number of erythroid lineage cells, a correlation between histopathological changes in the spleen and bone marrow and clinical pathology parameters revealed decreased RBC counts and a compensatory increase in reticulocytes (%). Although hemosiderin itself does not play a direct role in hematopoiesis, its presence indicates a history of RBC turnover. By functioning as an iron depot, hemosiderin facilitates the efficient storage and regulation of iron efficiently [39]. Given the pivotal role of iron in blood cell production and function, an adequate supply and storage are crucial for supporting hematopoiesis [40]. The antioxidant properties of iron contribute to robust hematopoiesis and cellular protection against oxidative stress [41]. Additionally, iron affects immune system function, with sufficient supply enhancing resistance to infections and diseases by supporting immune cell production and function.

No Observed Adverse Effect Level (NOAEL) for the 13-week repeat dose toxicity of HemoHIM G was 5000 mg/kg/day in SD rats. This supports the safety of HemoHIM G for daily use as a functional food.

## Data Availability

The datasets used and/or analyzed during the current study available from the corresponding author on reasonable request.
