# Correction: Evaluation of acute, 28-day, 13-week repeated dose oral toxicity and genotoxicity of a herbal extract (HemoHIM G) from *Angelica sinensis*, *Ligusticum chuanxiong*, and *Peaonia lactiflora*

**DOI:** 10.1007/s43188-026-00367-7

**Published:** 2026-10-06

**Authors:** Su-Bin Bak, Hansol Choi, Gyoung-Deuck Kim, Ju Gyeong Kim, Da-Ae Kwon, Ha-Young Kim, Dong-Won Son, Jang-Hun Jeong, Byung-Woo Lee, Hyo-Jin An, Hak Sung Lee

**Affiliations:** 1 Food Science R&D Center, Kolmar BNH Co., Ltd., 61, Heolleung‑ro 8‑gil, Seocho‑gu, Seoul, Republic of Korea; 2 Biotoxtech Co., 53, Yeongudanji‑ro, Ochang‑eup, Cheongwon‑gu, Cheongju‑si, Chungcheongbuk‑do 28115 Republic of Korea

**Correction: Toxicol Res. (2024) 40:297–311** **https://doi.org/10.1007/s43188-024-00227-2**

In section ‘Materials and methods’, subsection ‘Test substances’, the term ‘*Angelica gigas*’ should have read ‘*Angelica sinensis*’.

The original article has been corrected.

